# Why is health improvement policy so difficult to secure?

**DOI:** 10.12688/openreseurope.14841.2

**Published:** 2022-08-26

**Authors:** Paul Cairney, Emily St.Denny, John Boswell

**Affiliations:** 1 Division of History, Heritage, and Policy, University of Stirling, Stirling, Stirling, FK94LA, UK; 2 Department of Political Science, University of Copenhagen, Copenhagen, DK-1353, Denmark; 3 Politics and International Relations, University of Southampton, Southampton, SO17 1BJ, UK

**Keywords:** Policymaking, Public Health, Prevention, Health Improvement, Equity, Inequalities, Complexity

## Abstract

Many governments seek to improve population health, and some seek to reduce health inequalities. Yet, there remains a large gap between their policy statements and actual outcomes. Perennial questions in public health research include: why is this gap so large, why does it endure, and what can be done to close it? This essay uses political science and policy studies insights to address these questions, focusing on the distinctive issues that relate to (1) broad aims like ‘prevention’, (2) specific strategies for health improvement, or (3) new events. On the one hand, the idea of ‘prevention’ has widespread appeal, when governments think they can save money or reduce inequalities by preventing problems happening or worsening. While health protection seeks to inoculate populations against communicable diseases, health improvement strategies, including ‘Health in All Policies’ (HiAP), primarily address non-communicable diseases (NCDs). Further, the coronavirus disease 2019 (COVID-19) pandemic highlights the unequal spread of ill health, showing that preventive health ideas should be at the core of policy. On the other hand, there is a large gap between rhetorical and substantive commitment to prevention, a continuous HiAP implementation gap, and a tendency for COVID-19 health protection to overshadow health improvement. Explaining each problem clearly helps to identify the factors that *generally* undermine prevention policies and those *specific* to more detailed strategies like HiAP or events like COVID-19. We do not prioritise leadership or ‘political will’ as the policymaking problem. Instead, we identify the systemic factors that apply to even the most sincere, competent, and energetic policymakers. Health improvement policy is typically undermined by a lack of: *clarity* about what prevention means in practice; *congruity* between the prevention agenda (emphasising the need for major change to policy and policymaking) and routine government business; and, *capacity* to overcome obstacles to policy change.

## Plain language summary

We seek to explain a large and enduring gap between policymaker commitment to public health policies and actual outcomes. COVID-19 provides a new way to explain this old problem. COVID-19 should have prompted governments to treat public health as fundamental to public policy for two main reasons. First, many had already made strong rhetorical commitments to public health strategies, to prevent non-communicable diseases (NCDs, including heart disease, cancers, and diabetes) and address health inequalities. These strategies highlight the social and economic aspects of population health improvement which relate more to safe and healthy environments, education and employment, marginalisation, and economic inequality, than to healthcare. Second, COVID-19 highlights major health inequalities when the ability to stay safe from infection is not enjoyed by everyone, and there are strong links between NCDs and vulnerability to COVID-19 death or major ill-health.

Yet, COVID-19 responses side-lined health improvement. Health departments postponed health improvement strategies and moved resources to health protection.

This experience is important because it represents a new and extreme version of an old story of limited progress towards reducing health inequalities. It puts to rest the hope (expressed frequently within public health research) that the logic of health improvement is irresistible. Rather, there is an inevitable gap between health improvement goals and their implementation, it is difficult to generate momentum for health improvement strategies, and it can be lost at any time.

In that context, we explain why any new ‘window of opportunity’ for health improvement policies could be illusory. We encourage public health advocates to focus less on what they hope will happen (and what they need from policymakers) and more on what they can realistically pursue.

## Introduction

There is a profound and continuous gap between the rhetoric and practice of preventive policymaking. We use political science and policy theories to examine and inform the attempts by public health researchers and advocates to close it. We then use these insights to explore the future of preventive health in the context of COVID-19, focusing on health improvement (also known as health promotion) strategies to prevent non-communicable diseases (NCDs) such as heart and respiratory diseases, strokes, cancers, and diabetes.

The idiom ‘prevention is better than cure’ has rhetorical weight across many governments. It sums up the idea that governments can save money and reduce inequalities by engaging preventively, to stop problems before they happen or prevent them getting worse ( Kennedy, 2020). Policy initiatives backed by this general idea can be found in sectors including social policy, education, and criminal justice, and cross-sectoral initiatives such as ‘preventive spending’, and can be traced back to initiatives over the past century ( Cairney & St.Denny, 2020).

Further, prevention has particular resonance in public health. A focus on *health protection* offers the chance to prevent illness by inoculating populations against pandemics of communicable disease. A focus on *health improvement* offers the chance to improve the social, economic, and physical environment to prevent an epidemic of NCDs and improve health equity. For example, the approach known as ‘Health in All Policies’ (HiAP) sums up the latter: highlighting the ‘social determinants’ of health (and unfair health inequalities), describing health as a human right and the need to pursue health equity, and proposing high levels of cooperation across government to produce policies to address NCDs ( WHO, 2014). This focus on social or structural determinants challenges a tendency to relate health inequalities primarily to biology (‘there is no biological reason for their existence’, Whitehead & Dahlgren, 2006: 4) or individual ‘lifestyles’ in relation to healthy eating, exercise, and the avoidance of smoking and alcohol. It identifies the profound impacts on population health from (a) environments outside of an individual’s control, in relation to threats from others, such as pollution or violence, (b) education and employment, and (c) economic inequality, influencing access to warm and safe housing, high quality water and nutrition, choices on transport, and access to safe and healthy environments ( Bliss *et al.*, 2016: S88). It also warns against relating health improvement to health *care*, since most policy solutions are in non-health sectors ( De Leeuw & Peters, 2015).

Most recently, the COVID-19 pandemic has provided stark warnings of the importance of social determinants and health inequalities ( Bambra *et al.*, 2021). First, the prevention of NCDs is central to reducing the impact of COVID-19. Vulnerability to major illness and death relates strongly to heart disease, diabetes, and lung conditions ( Kluge *et al.*, 2020). Second, it highlights social determinants in relation to self-isolation and social distancing: some people have access to food, private spaces to self-isolate, and open places to exercise away from others; many have insufficient access to food, no private space, and few places to go outside; and, this ability varies widely within and across countries ( Shadmi *et al.*, 2020). There is mutually reinforcing harm from infectious disease, chronic disease, and ‘social conditions’ (often described as a ‘syndemic’, Bambra *et al.*, 2020: 965; Todd & Bambra, 2021).

If we combine these reasons to support prevention policies, wouldn’t we expect them to rise to the top of national government *and* international agendas and stay there until policymakers saw major improvements in population health outcomes? The answer from policy research is an emphatic *no* ( Cairney & St.Denny, 2020). Prevention generally receives only lip service, particularly among ‘neoliberal’ governments that emphasise personal responsibility and resist calls for major state intervention ( De Leeuw & Peters, 2015: 987–8; Oni *et al.*, 2019). HiAP studies highlight limited progress. COVID-19 and health protection knocked health improvement off the policy agenda in most countries ( WHO, 2020).

In that context, this Essay serves as a way to step back, reflect, and comment on what we know about these enduring policy problems, based on insights from political science and policy process literatures. We ask three separate questions to highlight the relationship between the factors that *generally* undermine prevention policies (not only in health) but may arise in *specific* ways in relation to health improvement strategies such as HiAP or government responses to COVID-19.

Section one identifies how to explain the general lack of prevention policy. Generally speaking, why is there such a gap between rhetorical commitment to the broad idea of prevention versus actual policies, practices, and outcomes?

Section two connects broad insights on prevention to the specific study of health improvement strategies such as HiAP. If they are designed to help overcome such problems, why is there still a large and continuous implementation gap?

Section three explores what additional problems are associated with COVID-19 policies, in which a focus on health protection overshadowed and undermined health improvement ( WHO, 2020). It uses insights from sections one and two to inform research and practice, focusing on the future of public health policies: what will happen when governments disinvest in their COVID-19 emergency responses and return to a greater focus on health improvement? In public health research, it is common to cite one political science perspective – Kingdon (1984) – to signal that a new ‘window of opportunity’ for health improvement policy may open. However, sections one and two provide cautionary tales to underpin future strategies. They show that vague political agreement – to mainstream health across government – is no guarantee of substantial action, and the production of yet another new strategy is futile without knowing how consistent it will be with routine government business. The window of opportunity may open to produce a half-baked solution to an ill-defined problem.

This three-stage focus helps to identify how policymakers and practitioners can draw lessons on prevention policy and health improvement during the absence and presence of crises. What can they learn from each experience to help address this prevention puzzle?

## Methods and approach

This Essay employs narrative synthesis to identify general and specific lessons for research and practice. Section 1 synthesises insights from our previous work that applied political science and policy theories to multiple case studies of prevention and public health policies (including Boswell, 2016a; Boswell 2016b; Boswell *et al.*, 2019; Cairney *et al.*, 2019; Cairney, 2020; Cairney & St.Denny, 2020). We generate a simple framework that categorises common obstacles to policy and policymaking reforms, using alliteration – describing *clarity*, *congruence*, and *capacity* – to aid the narrative. Section two connects these broad insights to the qualitative systematic review of HiAP studies that Cairney and St.Denny co-authored (with Heather Mitchell) in *Open Research Europe*. The detailed account of our systematic review methods, reproducibility, ethics, and further data can be found in Cairney *et al.*, 2021b. Here, we focus on its take-home messages, which we use in section three to narrate key issues of policymaking in the COVID-19 era.

The final part of our approach is to seek feedback from practitioners on the usefulness of this essay when considered in different contexts, and to compare our insights with theirs. To that end, our first step was to share insights in an academic-practitioner workshop in Scotland, in May 2022 (summarised in Cairney *et al.*, 2022a). Our aim is to use the flexibility of the *Open Research Europe* platform to prompt further reflection and update the Essay in line with future workshop activity.

## Section 1. The general problem with prevention policies: explaining the gap between rhetorical commitment and actual practices and outcomes

Prevention is a recurrent theme in efforts to improve policymaking and respond to complex policy problems. It relates to issues that cut across policy silos and sectors, to tackle problems like inequalities ( Cairney & St.Denny, 2020), climate change ( Bradford *et al.*, 2018), social deprivation ( White, 2017), and criminal justice ( Sherman & Eck, 2002). It travels across levels of policymaking, in the documents of the WHO ( Mendis, 2010), EU ( Mackenbach, 2006), national governments ( DHSC, 2018; National Preventative Health TaskForce, 2009), and subnational governments ( Craig & Robinson, 2019; Haynes *et al.*, 2020). Its logic is boosted by idioms and metaphors: ‘prevention is better than cure’; ‘a fence at the top of the cliff is better than an ambulance at the bottom’ ( DHSC, 2018; Shallowe *et al.*, 2020). It links closely to ‘futures thinking’, ‘horizon-scanning’ or ‘anticipatory governance’ ( Guston, 2014; Kuosa, 2016; van Rij, 2010). Almost everyone sees prevention as an idea worth pursuing. Yet, actual practices and outcomes fail to match this rhetoric, contributing to a cycle of enthusiasm in theory and despair in practice ( Cairney & St.Denny, 2020).

A synthesis of political science and policy studies insights helps to explain such dispiriting outcomes. These explanations begin with general reference to two foundational concepts ( Cairney, 2020; Cairney *et al.*, 2022b). First, *bounded rationality* describes how policymakers deal with the limits to their ability to process information and make choices. They draw on cognitive and organisational shortcuts: ‘rational’ ways to prioritise trusted sources of information, and ‘irrational’ ways to draw on their gut instincts and emotions to draw quick conclusions ( Cairney & Kwiatkowski, 2017). Health studies respond to this dynamic by promoting ‘evidence based policymaking’ and generating more information to reduce policymaker uncertainty ( Cairney, 2016). Political science accounts highlight the importance of ambiguity: there are many ways to define or ‘frame’ a policy problem, prompting policy actors to exercise power to draw policymaker attention to their preferred framing at the expense of others, and generating demand for evidence in that context ( Cairney *et al.*, 2016).

Second, *policymaking complexity* describes the environments over which policymakers have limited knowledge and even less control, summed up with reference to five concepts:

1. *Actors*. There are many policymakers and influencers spread across many levels and types of government (otherwise known as *venues* of authoritative choice).
2. *Institutions*. Each venue has its own rules and norms. Formal rules are written and well-understood. Informal understandings are unwritten and often communicated non-verbally.
3. *Networks*. Elected policymakers delegate most policy to bureaucrats, who seek information and advice from interest groups and experts. Policymakers and influencers trade access for advice, forming networks that often exclude most other actors.
4. *Ideas*. Each venue or network operates with reference to well-established beliefs about the nature of the policy problem and the feasibility of each solution.
5. *Context and events*. Policymakers must respond to socioeconomic factors and crises that are not in their control ( Cairney *et al.*, 2019: 6–7).

These concepts inform studies of: (1) *multi-centric policymaking*, in which policymaking power is spread across many venues rather than concentrated in a single core executive, (2) *governance* rather than government, in which policymaking is often characterised by blurry boundaries between those who make, influence, and deliver policy, and (3) *complex policymaking systems*, in which policy outcomes seem to ‘emerge’ in the absence of central government control and ‘implementation gaps’ are routine features, not bugs (2019: 7; see Harris *et al.*, 2014; Harris *et al.*, 2015 for applications in public health policy).

We use these insights to identify three causes of the prevention puzzle. First, there is a continuous lack of *clarity* about what prevention means. People may agree on broad aims in principle because they are ambiguous, but this consensus disappears when they have to produce concrete responses. Second, there is low *congruity* between preventive aims and policymaking-as-usual. Debates on concrete responses reveal that preventive policies could involve radical changes, such as to use tax and spending to promote a major redistribution of resources, produce far greater state intervention to influence business or individual behaviour, or reform policy processes to encourage ‘joined-up’ policymaking. Some actors tend to take these proposed changes for granted, while others tend to think they were signing up to more modest proposals. Third, there is low *capacity* to deliver radical changes to policy or policymaking in the face of many obstacles. When policymakers understand what it would take to challenge the status quo to produce new policy outcomes, few are willing to do so. However, they still use the general language of prevention. Consequently, the continuous appearance of rhetorical support for preventive policies masks the lack of substantive support to deliver.

### Clarity: if prevention means everything, maybe it means nothing

Prevention can be superficially attractive to policy actors, but for different reasons and with different assumed implications. The ambiguity of ‘prevention’ initially allows people to attach very different meanings to the same language. Actors can use it to define very different problems, including the need to reduce public sector costs or health inequalities, or different solutions, representing radical or modest changes to current policies. Initial ambiguity also helps to establish agreement without surfacing tensions. As a result, the competition to define prevention – in relation to problems or potential solutions – is not immediately obvious. Some actors or professions may feel that *they* know what it means in their context, but this narrow agreement is of limited value if not shared by actors making key choices. At some point, this competition needs to happen. Indeed, politics is about a much wider power dynamic involving cooperation and competition to establish meaning by ‘framing’ issues in ways that favour some actors at the expense of others ( Baumgartner & Jones, 2009).

For most of its vocal proponents in academia, prevention and health improvement is associated with equity ( Marmot, 2005): state intervention should focus on re-drawing the social landscape, to support those from vulnerable or marginalized backgrounds *before* their disproportionate disadvantages begin to accrue and compound. Yet, not everyone shares this focus on inequalities (in government or health research). Other supporters of prevention have understandings conducive to much smaller, more targeted, and incremental sorts of policy change. This distinction often relates to an unclear ecological metaphor: upstream measures seek to change the social environment, while midstream and downstream measures target companies or mitigate the effects of the environment on at-risk groups or individuals ( McMahon, 2021a; McMahon, 2021b).

For example, Boswell’s (2016a) study of political debate on obesity in Australia and the UK uncovers competing narratives that lie beneath superficial consensus on the value of prevention. For some advocates, prevention would mean engaging in radical policy and policymaking changes, seeking (1) fundamental socio-economic transformation to alleviate the social inequalities that drive obesity, and/ or (2) the strict regulation of the production and marketing of food to mitigate ‘obesogenic environments’. Outside this world of public health, moderate ideas enjoy greater currency. Support for preventing obesity typically means investing in medical interventions, or social marketing campaigns to encourage populations to make healthier choices. The shared commitment to prevention in principle eventually gives way to an intense but often hidden political contest over the meaning in practice.

This temporary agreement is a recurrent feature across the many settings, sectors and issues for which prevention holds so much appeal. Policy research shows that ambiguity provides a valuable resource to policymakers as it enables widespread buy-in for general ideas (see Yanow, 1996; Zahariadis, 2003). In practice, low-profile contests over the meaning of prevention generally get resolved in favour of minimal policy change ( Cairney & St.Denny, 2020).

### Congruity: prevention is out of step with routine government business

Ambitions for major changes to policy and policymaking flounder when they are incongruous with ‘business-as-usual’ policymaking. A wide variety of terms exist to describe causes of policy inertia in policymaking systems, including ‘inheritance before choice’ (the first act that new governments make is to accept the policy commitments of their predecessors), ‘policy succession’ (most ‘new’ policies are actually modifications of old programmes), and ‘path dependence’ (choices made in the past contribute to the rules and practices that still govern the present) ( Hogwood & Peters, 1983; Mettler & SoRelle, 2018; Pierson, 2000; Rose, 1990). They all suggest that, while governments find the language of radical policy change useful, it does not really sum up what they do in practice. This is particularly true when the adoption of radical prevention initiatives relies on a ‘leap of faith’ ( Botterill, 2006) that policymakers are unwilling to take, or when those initiatives would present a challenge to their higher salience aims (such as to prioritise particular economic models, or high funding for healthcare). Major policymaking reforms are difficult enough when everyone generally agrees on what to do, and present often-insurmountable obstacles when aims and outcomes are contested.

The first obstacle is political short-termism. The promise of prevention is in long-term and uncertain outcomes. However, most decisions are made on a short timeframe between elections, which focuses attention on more immediate and tangible costs and benefits ( MacKenzie, 2016). Or, the urgent crisis today crowds out the potential crisis tomorrow ( Hammond & Smith, 2017; Mazey & Richardson, 2021; Rhodes, 2011; van Dorp & Hart, 2019). The most recent example is COVID-19, in which the urgent needs of health protection have enabled massive intervention often at the expense of hard-fought efforts to enable health improvement and tackle the long-term drivers of chronic disease ( WHO, 2020).

The second obstacle is the bureaucratic politics largely hidden from public view. In some narratives, there are clear intentions to reproduce the status quo. Here, we see how institutions – ‘the way things work around here’ – are reinforced by the networks of actors that influence, make, and deliver policy ( March & Olsen, 1984; Rhodes *et al.*, 2006; Schmidt, 2010; Streeck & Thelen, 2005). These rules, norms, and routine practices help mollify intervention. Behind the scenes, powerful actors use these rules to thwart radical action ( Hawkins & Holden, 2014; Miller & Harkins, 2010). In other narratives, these dynamics relate to complex policymaking systems, in which the interaction between many actors – reproducing a multiplicity of (often contradictory) rules – can undermine new initiatives regardless of intent ( Cairney, 2020: 104-7).

Consequently, there are regular efforts to ‘institutionalize’ prevention in policymaking: creating organizational structures or dedicated agencies within government to offer leadership and challenge short-termism and delay ( Boswell *et al.*, 2019; Smith, 2020). In practice, these agencies account for a tiny proportion of a sector’s budget and frequently fail to disrupt policies and outcomes, with powerful rival organizations in and out of government rendering their status precarious ( Cairney *et al.*, 2021b: 15). The radical nature of prevention makes it difficult for agencies to establish credibility and value, especially among the actors whose practices they challenge, and when the evidence for the impacts of interventions are hard to demonstrate during normal policymaking timelines. They become marginalized by the ‘business-as-usual’ practices of evaluation and accountability in policymaking.

### Capacity: low support for major investments with uncertain rewards

Capacity can relate to *agency*, to describe the limited ‘will’ to pursue policy change, or *context*, to describe the limited resources that governments have to coordinate policy change in systems beyond their control. The latter can result from *choice*, to restrict the role of the state or share responsibilities, but also *necessity*, to reflect the limits to central control that we describe at the beginning of Section one ( Cairney *et al.*, 2019: 6–7). These factors help to explain why policymakers frequently conclude that many government reforms are not feasible in relation to the immediate constraints in which they take decisions. Indeed, the predominant story about policy capacity, especially in liberal democracies, is of state retrenchment and increasing reliance on third and private sector actors to make and deliver policy ( Hardiman & McCarthaigh, 2017; Wu *et al.*, 2015).

Preventive policymaking has some additional problems with capacity. First, the common perception is that many preventive measures are prohibitively costly with no immediate payoffs. Preventive measures might be economical on long-term timescales; indeed, that is their founding logic, regarded as ‘self-evident’ in public health ( Capewell & Capewell, 2018). However, the immediate reality is of tight budgets and limited bureaucratic capacity. New initiatives struggle to win support, given the tendency of governments to see their current balance of taxing/spending as not for negotiation. Even interventions with a history of investment and widespread legitimacy can face budget cuts.

Second, there is often a lack of capacity or commitment to overcome major opposition to policy change. Government coordinative capacity is limited when relying heavily on governmental and non-governmental organisations to make and deliver policy. In theory, they could combine delegation with strong regulation, but in practice most governments tend towards a ‘hands off’ approach (partly to help avoid blame for policy outcomes, Cairney *et al.*, 2019: 9; 24–5). Further, when preventive measures entail ‘hard choices’ that impact on powerful industries, governments prefer to persuade than compel ( Godziewski, 2022).

These general and specific limits combine in different ways. For example, the Sure Start early intervention programme in the UK was initially treated as a high-priority and successful programme to detect and address social, health and educational issues in families. However, the cross-cutting nature of ‘families policy’ presented general problems of coordination across multiple departments (most of which did not treat it as a priority), and it became difficult for the Labour government to measure and describe its success in ways that were conducive to long-term political support ( Cairney & St.Denny, 2020: 93-8). Indeed, it was then scaled back as part of the austerity measures of the new Coalition government in 2010 ( Torjesen, 2016).

This kind of experience differs from the more contested arenas of public health – such as tobacco, alcohol, and food policies - in which there is clearer opposition to state intervention. In such cases, industry actors are relative experts at exerting influence throughout the policy process, and enjoy a particular advantage in impacting the low-profile institutions and practices that turn vague agreement (on prevention as a goal) into tangible action ( Boswell, 2016b). The classic examples are voluntary or self-regulated compliance schemes for industry, which symbolize low government commitment and capacity to enforce policy change ( Baggott, 1986; although examples such as tobacco control show the potential to shift towards regulation – Cairney & St.Denny, 2020: 149–53). Global trends highlight a shift to asking food companies to restrict and monitor marketing of unhealthy products to children, and alcohol and sports betting companies to fund social marketing on responsible drinking and gambling. However, public health actors are naturally sceptical of the claims made for these schemes (often based on their cynical use in the name of tobacco control, Cairney *et al.*, 2012). Most evidence suggests that these initiatives fail to deliver on their purported ‘efficiency’ benefits of working with, rather than regulating, industry ( Hastings, 2012; Lacy-Nichols *et al.*, 2020). Nevertheless, their prevalence speaks to the broader point: that policymakers feel reliant on private sector actors and prefer to engage in collaboration than outright hostility ( Boswell *et al.*, 2019; Godziewski, 2022).

***The triple threat of low clarity, congruity, and capacity.*** The general dynamic of prevention policy suggests that vague rhetorical commitment gives way to power and politics in policymaking systems that defy control. A lack of *clarity* about the meaning of prevention enables initial buy-in and momentum from a broad coalition of actors. However, these actors have incommensurate ideas about what prevention ought to entail. The more ambitious and radical hopes of public health researchers and practitioners tend to get dashed as this ambiguity is thrashed out in policy work. These hopes tend to lack *congruity* with ‘business-as-usual’ in comparison with more moderate solutions. Governments also feel they lack *capacity* to invest in ambitious and radical policies, or – in many cases – to tackle powerful industry actors head-on. The result is a perpetuating cycle of enthusiasm and frustration, where bright ideas and good intentions founder on the mundane realities and constraints of policymaking ( Cairney & St.Denny, 2020).

## Section two. The problem with health improvement strategies: explaining the ‘implementation gap’ in Health in All Policies

This general story provides much-needed context during the study of Health in All Policies (HiAP). If we know that vague commitment and high aspirations tend to translate into unfulfilled expectations, we can *explain* limited progress. Yet, health improvement advocates seek also to *overcome* such obstacles, to see HiAP as an ambitious and highly supported strategy, containing a coherent narrative, and supported by (what Cairney *et al.*, 2021b call) a ‘playbook’ to turn strategy into action. If so, unlike the vague concept of prevention, HiAP strategies appear to have more substance. A government commitment to HiAP comes with a model and set of instructions to carry it out. In that context, we might expect more progress since there appears to be more clarity and commitment. However, Cairney *et al.*’s (2021b) review shows that HiAP encounters very similar problems. First, it is not as clearly defined as it first appears and its playbook (or list of actions to foster high commitment and cooperation) does not help to deliver improved health equity. Second, researchers frequently bemoan a temporary ‘implementation gap’ which may be better interpreted as the routine consequence of governance in complex policymaking systems.

### HiAP and clarity

Cairney *et al.*’s (2021b: 6–8) review highlights the frequent claim (in public health research) that HiAP does not suffer from vague commitment since it is built on a powerful and coherent narrative with specific implications:

1. ‘Treat health as a human right’ (2021: 6).
2. “Identify evidence of the ‘social determinants’ of health inequalities” (2021: 6).
3. “Identify evidence-based ‘upstream’ solutions” to address and mitigate inequality as early in people’s lives as possible (2021: 7).
4. ‘Promote intersectoral action and collaborative governance’ to ensure a coherent response across (and outside of) government, recognising that most impacts on health are not in the control of health departments (2021: 7).
5. ‘Seek high and enduring political commitment’ to ensure health equity remains high on the agenda and gains resources for implementation (2021: 8; see also Bharmal *et al.*, 2015; Carey & Crammond, 2015; *Helsinki Statement on Health in All Policies*, 2014; Williams *et al.*, 2008).

This narrative underpins the WHO (2014: 12–17) ‘starter kit’ to generate demand for a HiAP strategy, make it feasible in each political context, identify which actors and processes will aid progress, build capacity, promote Health Impact Assessment (HIA), and monitor progress towards health equity. Further, Cairney *et al.* (2021b: 8–13) show that this starter’s kit informs a wider collection of commonly-dispensed advice on how to foster HiAP success (which they describe as the HiAP ‘playbook’):

1. ‘Use well-established ways – such as by using the WHO starter’s kit - to get from talk to action, and to sustain long-term commitment’ (2021: 8).
2. ‘Raise awareness and connect HiAP to a government’s values and policy agendas’ (2021: 8). For example, frame HiAP aims to be consistent with a government’s overall vision and useful to its core business.
3. ‘Focus on win-win solutions to foster trust-based intersectoral action’ (2021: 9). Generate the sense that HiAP provides mutual gains between health and other sectors, such as building trust and establishing the payoffs to collaboration.
4. “Avoid projecting a sense of ‘health imperialism’” (2021: 10). Avoid the sense that HiAP represents interference in non-health sectors – undermining their core business and provoking reactions against HiAP – by contributing to shared agenda across sectors.
5. ‘Identify policy champions and entrepreneurs’ (2021: 10). Key actors use their knowledge, networks, and skills to kickstart HiAP and ensure regular progress.
6. ‘Use HiAP to promote the routine use of HIAs’ (2021: 10). HIAs are formal processes to help identify the value of non-health initiatives to reducing health inequalities.
7. ‘Do not rely on a traditional cost-benefit-analysis case for HiAP’ (2021: 11). Find ways to establish the value of HiAP that do not rely on a narrowly defined economic case.

Yet, Cairney *et al.* (2021b: 12–20) find that almost every relevant HiAP study describes (from the perspective of HiAP researchers) a dispiriting ‘implementation gap’ that is not overcome by the playbook. In practice, HiAP encourages considerable wriggle room as specialists interact and collaborate with other policy actors. Cairney *et al.*’s (2021b: 12–20) reflections on empirical studies suggest that HiAP is too abstract to translate into action in a straightforward way ( Huang *et al.*, 2019: 2). Rather, its meaning and definition can vary markedly ( Storm *et al.*, 2014: 184). As such, ‘every HiAP initiative is uniquely designed and governed, and so it is challenging to understand how to translate studies of one case to others’ ( Shankardass *et al.*, 2018: 2). Indeed, Cairney *et al.* (2021b: 12–20) find that almost all of the terms essential to the meaning of HiAP remain ambiguous, including ‘social determinants of health’, ‘upstream’ measures, collaboration, ‘political will’ ( Baum *et al.*, 2020; Carey & Friel, 2015; de Leeuw, 2016; O’Flynn, 2016; Post *et al.*, 2010). Therefore, any sense of coherence would be misplaced, contributing to confusing advice on how to operationalise and deliver HiAP aims.

### HiAP and congruity

Many HiAP advocates have tried to ensure congruity with policymaking norms and routines. A key focus has been on framing HiAP as consistent with a government’s overall vision and useful to its core business (playbook point 2), in the hope that HiAP becomes mainstreamed throughout government policy and an accepted way to judge performance ( Freiler *et al.*, 2013: 1070; Greaves & Bialystock, 2011: 407; Molnar *et al.*, 2016: 2–3). Examples include framing HiAP as: a way to reduce the unsustainable burden on health services ( Kickbusch *et al.*, 2014: 187–8); ‘stimulating economic productivity’ ( Delany *et al.*, 2016: 888); and, essential to ‘EU core values such as solidarity, equity and universality’ ( Bert *et al.*, 2015: 45).

However, these efforts have enjoyed limited success, especially when tensions accrued across policymaking levels and silos. For example, HiAP studies show there are significant risks and downsides to top-down approaches preoccupied with alignment with central government, largely because much of the work of public health takes place in local settings with established norms, practices and relationships among networks of actors. This ongoing and unresolved tension between top-down and bottom-up approaches to governance is a general feature of policymaking ( Cairney, 2022) and it arises frequently in HiAP reports, partly because HiAP strategies accommodate rather contradictory aims pursued by different actors. We see this especially in potential best-case exemplars in Nordic countries. Despite traditions of decentralized governance and a strong welfare state, the experience in Nordic countries offers a cautionary tale. New initiatives and organisational forms driven from above have met with ambivalence or resistance as local actors – often excluded from the initial development of strategies – grapple with complex and often contradictory demands ( Hagen *et al.*, 2018; Melkas, 2013; Scheele *et al.*, 2018; Synnevåg *et al.*, 2018).

Cairney *et al.* (2021b) identify a large number of studies which highlight limited progress in relation to overcoming siloed, sectoral conflicts. Key issues include the sense of ‘health imperialism’ that arises when ‘problems and the necessary actions are defined from the viewpoint of the health sector only’ ( Delany *et al.*, 2016: 889; see also Breton, 2016: 383–4). A perception of external interference can lead to defensive interactions, poor collaboration, jurisdictional conflicts, and opposition to the extra health-focused work that comes at the expense of current commitments ( Gottlieb *et al.*, 2012: 158; Guglielmin *et al.*, 2018: 287–90; Lawless *et al.*, 2012: S15; Newman *et al.*, 2014: 54; Oneka *et al.*, 2017: 836; Synnevåg *et al.*, 2019: 7).

HiAP proponents have responded with what they describe as a *win-win* approach (playbook 3), based on a ‘shared vision across sectors’ ( Guglielmin *et al.*, 2018: 291), or the sense that HiAP can help other actors deliver their core aims ( Freiler *et al.*, 2013). Others have promoted a new ‘shared language’ across sectors ( Molnar *et al.*, 2016: 8–10); rebranded HiAP aims in terms of wellbeing, ‘living conditions’, ‘social sustainability’, ‘human rights’ or ‘civic participation’ to generate cross-sectoral ownership ( Scheele *et al.*, 2018: 64; Synnevåg *et al.*, 2018: 70–1); or rebranded Health Impact Assessment (HIA) as ‘overall policy appraisal’ ( Kemm, 2001: 82–4).

Yet, in practice, these cross-sectoral gains are not easily won, nor sustained over time. Without careful deliberation and engagement to sustain buy-in, HiAP strategies and initiatives still trigger professional-identity-driven opposition to ‘health imperialism’ (despite attempts to address this problem in playbook 4). Further, such problems with informal ways of working are not solved by formal reorganisations. Holt *et al.*’s (2018) account of HiAP in Danish municipalities concludes that sectoral re-organisations ‘tend to reproduce the organizational problems they are intended to overcome’, suggesting that ‘It is time to dismiss the idea that intersectoral action for health can be achieved by means of a structural fix’ (2018: 48).

### HiAP and capacity

There have also been efforts to build capacity to ensure sustainability for the HiAP agenda. Proponents have tried to establish a credible track record for HiAP initiatives, and to generate strategic support at a high level. One focus has been on establishing a robust and legitimate toolkit for analysis and evaluation, with HiAP as the driver and processes such as HIA (playbook 6) as contributors. This has meant challenging the value of simplistic cost-benefit approaches to understand overall costs, return on investment, or efficiency (playbook 7). Advocates understand it is important to demonstrate the economic value of HiAP, but hold that it is difficult to make a short-term ‘business case’ because:

‘(1) public health benefits are generally dispersed and delayed; (2) benefactors of public health are generally unknown and taken for granted; (3) the costs of many public health initiatives are concentrated and generate opposition from those who would pay them; and (4) public health often clashes with moral values or social norms’ ( Mayes & Oliver, 2012: 181).

It has proven challenging to argue that the HiAP business case requires different rules to justify economic investments ( Pinto *et al.*, 2015: 2–6). Rather, a pragmatic option has been to accept minimal additional funding for HiAP, and seek to incorporate it into existing budgets. This strategy comes with the risk of preventive policies being treated as expendable, particularly during austerity or if multiple levels of government fund the same project (2015: 4–5).

Another focus has been on identifying champions for the HiAP agenda (playbook 5). HiAP studies describe the decisive impact of key individuals able to use their resources to address multiple obstacles to HiAP progress. Some describe case studies of individuals ( Bliss *et al.*, 2016: S91; Kickbusch *et al.*, 2014: 187–92). Others focus on developing the skills to perform key roles ( Damari & Chimeh, 2017: 407; Hendriks *et al.*, 2014: 175).

Yet, even high-level buy-in for HiAP has not translated into tangible action. We can take the best-case scenario of high-level, centralised buy-in - South Australia (SA) - as another cautionary tale. SA exhibited strategic buy-in for HiAP within the central Department of Premier and Cabinet and investment in a unit to promote HiAP policies and programmes. Yet critical reviews (especially Delany *et al.*, 2016) reveal that the reality has not matched the rhetoric, with the commitment to HiAP undermined by the changing political fortunes of prominent champions and an overarching context dominated by norms of small government, austerity and state retrenchment.

In other words, the general conclusion from Cairney *et al.*’s (2021b) review is that HiAP capacity is elusive, regardless of the strength of official rhetoric. In that context, HiAP studies describe an inescapable tension between HiAP aims. One is to adapt to existing practices while encouraging new solutions ( Bowman *et al.*, 2012: 847). Another is to be more challenging: ‘speaking truth to power’ to overcome the business-as-usual approaches and dominant ways of thinking in government that would otherwise thwart HiAP progress ( Carey & Crammond, 2015: 1026).

### Reflections on the HiAP experience

Cairney *et al.* (2021b: 27–8) conclude that HiAP ‘has proven to be a vague proposition backed by an ineffective playbook’. Their general discussion of country studies identifies a major gap between HiAP as a broad strategy and the actual policy outcomes that it is used to deliver. They note that *even the best-case examples* highlight major problems. The Nordic country experience suggest that their contexts – a high commitment to extensive welfare states and to the meaningful sharing of responsibilities between national and subnational governments – should be conducive to HiAP aims. The South Australia experience – built on high political commitment at a strategic level – should provide a new impetus for policy change. Yet, in each case, this appearance of a supportive context dissolves in practice. Nordic experiences highlight central-local tensions and varying commitments to delivering HiAP aims. South Australia exemplifies a tendency for HiAP commitment to be “overshadowed by ‘neoliberal’ policymaking, state retrenchment, and a commitment to protect reactive health services” (2021: 28). As a result, they reflect that:

‘the dominant narrative of HiAP *in theory* does not correspond to the meaning of HiAP *in practice*. The former is an ambitious strategy to address the social determinants of health with radical policy change across multiple sectors, facilitated by intersectoral action and high strategic commitment to produce support for better policies. The latter is an ambitious strategy on paper only, representing moderate policy change at best and a negative commitment at worst, particularly when the funding and allocation of staff is minimal in relation to the wider sector.’ (2021: 28).

In other words, there only appears to be high commitment to HiAP when we view that commitment in isolation. It does not seem so impressive when we relate it to a much larger commitment to the status quo, in which there is no additional commitment to economically redistributive policies, or to boost welfare state provision, and a high and enduring commitment to treating highly-funded healthcare as the main solution to health inequalities. Further, a vague long-term commitment to fostering preventive population health and wellbeing approaches does not compete well with specific and short-term commitments to maintain reactive services. In that context, the idea that intersectoral action could help overcome the status quo is unrealistic. Indeed, the veneer of collaboration across and outside of government ‘helps *dilute* the ambitions of HiAP enthusiasts, not produce policies they favour’ (2021: 28).

## Section three. The new COVID-19 problem: explaining its additional negative impact

All of these HiAP studies were written pre-COVID-19. However, building on Cairney *et al.* (2021b), we argue that this new policymaking dynamic is impossible to ignore, not least because it adds a new twist to the HiAP narrative, with implications for health improvement more generally.

First, *COVID-19 should have prompted governments to treat health improvement as fundamental to public policy.* Many had made rhetorical commitments to public health strategies to prevent NCDs, and COVID-19 reinforces this rationale. Social determinants relate to health improvement (health inequalities resulting from factors such as income and social and environmental conditions) *and* health protection (unequal resources to live and work safely). Further, COVID-19 had a visibly disproportionate impact on the mortality and health of people with underlying health conditions associated with NCDs ( Bambra *et al.*, 2021). Second , *the opposite happened:* health departments – in many countries - postponed health improvement and moved resources to health protection ( WHO, 2020). Third, *these events are particularly dispiriting since attention to one aspect of public health comes at the direct expense of the other*. Practitioners in health improvement are used to dealing with slow progress in relation to fostering cooperation with *other sectors* and being undermined by *other agendas* (usually economic, relating to ‘austerity’ or ‘neoliberal’ policies) ( Cairney *et al.*, 2021b). In this case, they or their colleagues have been obliged to contribute to the reduced status of health improvement by shifting their energies to protection.

In that context, experiences from prevention policy provide a profound cautionary tale for the future. Many governments will be ‘rebooting’ or rethinking their health improvement strategies for a post-COVID world, and health improvement advocates will return to the language of ‘windows of opportunity’ for progress and the strategies summed up by the HiAP playbook. Yet, these approaches have not served them well, to the extent that it is worth stepping back to reflect on their future impact.

Cairney *et al.’*s (2021b) review of HiAP studies suggests that there is limited internal learning from experience thus far. Most proponents still treat policymaking as a technical exercise and use policy theories instrumentally, to find the right language to define the problem, the solutions that work, and the right model of intersectoral action and implementation. In other words, the alleged solution is to refine the playbook. This approach continues to underestimate the impact of politics on policy, in favour of functionalist arguments: identifying which policies *should* be selected, and how policymaking *should* work, rather than what actually happens ( Cairney *et al.*, 2022b).

An alternative is to take politics and policymaking complexity more seriously, reflecting on experiences so far, and identifying ways to take them into account. These processes of reflection, analysis, and learning should be guided by a clear map of the challenges which are: (1) relatively general features of policymaking and delivery, and (2) more specific to health improvement ambitions and principles.

### Getting clarity on what health improvement means in the COVID-19 era

Clarity can make the difference between minimal and maximal policy change. Advocates should not take for granted that the concrete measures required to put into practice the programmatic ideas associated with health improvement will flow naturally from initial commitments. Indeed, *the opposite may be true*. Securing high levels of national and international *in-principle* commitment to a vague idea can represent an *alternative* to substantive change ( Cairney & St.Denny, 2020).

The challenge lies in clarifying what a health improvement strategy reboot might look like. For example, does it come with a detailed plan of action with a starter’s kit and playbook or represent a more general ‘preventive philosophy’ for government? Is it a vehicle for the relatively top-down implementation of specific interventions, or to encourage collaborative governance in which health actors play an equal role? It is always tempting to answer ‘yes’ to all aims, but policymakers resolve ambiguity by making specific choices with resource implications.

If so, securing *in-practice* support for measures to redistribute resources, refocus policy horizons beyond the short-term, and bind sectorally disparate actors to common goals, represents a second level of policy change that needs to be negotiated and secured. Yet, as with most preventive policymaking initiatives, the HiAP literature contains a major internal contradiction that undermines this progress ( Cairney *et al.*, 2021b). Frankly, many HiAP advocates want to take control to produce policy instruments with specific ends (to reduce health inequalities) *and* give up control to work collaboratively with policy actors across and outside of government. They want to encourage collaboration to generate widespread ownership of policy change *and* reserve the right to reject the outcomes as not conducive to the HiAP agenda. In that context, if it is understandable to criticise *policymakers* for using ambiguity to avoid specific commitments, it is also reasonable to challenge health improvement advocates to address the ambiguity inherent in their approach.

### Enhancing congruity between health improvement initiatives and the ‘new normal’

Key elements of health improvement seem particularly vulnerable to disconnection with established practices and routines of policymaking. By definition, a radical policy agenda is out of step with the way things are done. The short-term pressures of the pandemic response have also seen governments strip back resources for longer-term health improvement. Recovering that knowledge, impetus and infrastructure will be a much harder feat than before, even if there is a ‘window of opportunity’ to do so. Crucially, the congruity challenge follows directly from a preferred definition of health improvement strategy based on two competing options.

First, if advocates see HiAP primarily as a global agenda to be adopted in similar forms in new domestic contexts, congruity is about altering radically the policies and structures of governments. If so, Cairney *et al.*’s (2021b) review identifies two congruity challenges that may never be overcome: specific HiAP units are too small and uninfluential to generate coordinative capacity, challenge business-as-usual government, or encourage new and sustainable ways of working; or, the formal reorganisation of coordinative mechanisms exacerbates coordination problems at the expense of more effective informal collaboration measures.

Second, if HiAP is a way to encourage intersectoral action and ‘collaborative governance’ ( Ansell & Gash, 2008), congruity is about playing one small part in a policymaking project. Policy scholarship describes essential practices, including: (1) incorporating formulation and implementation challenges into policy design; (2) embracing collaboration in policy design and implementation; and (3) understanding that ‘implementation’ is an often-misleading term, since policy often ‘emerges’ in the absence of central control, requiring adaption, learning, and persuasion to respond effectively ( Ansell *et al.*, 2017; Crowley *et al.*, 2020: 141–162).

### Building capacity to sustain the health improvement agenda

The goal of self-sustaining capacity to deliver health improvement needs to be grounded in policymaking reality, as described by studies of public administration and policy theory-informed empirical studies. The former identify how to foster intersectoral action, such as when Carey & Crammond (2015: 1022–8; see also Greer & Lillvis, 2014) describe a supportive governance ‘architecture’, skilful and flexible leadership, a manageable number of aims, and a powerful narrative to represent a common purpose. The latter explain the limits to coordinative capacity, including the strong logic for policy specialisation and silo working, and a tendency for the distribution of policymaking responsibilities to relate weakly to the task. In other words, it is unrealistic to expect all actors to come together to produce ‘coherent’ policies and practices ( Cairney *et al.*, 2021a; Cairney *et al.*, 2022). Intersectoral, collaborative, ‘mainstreaming’ initiatives *require* different actors spread across numerous sectors and operating at different levels to ‘pull together’ to deliver a multitude of complementary services. However, it would be a grave mistake to equate functional requirements with actual policymaking.

We have seen major cross-sectoral coordination activities in the crisis response to COVID-19, but these are short-term arrangements. Meanwhile, the formal architecture and informal relationships that existed for joined-up governance on health improvement have been de-funded and de-prioritised. Building back capacity for health improvement represents an acute governance problem, to harness the complex political and organisational systems required to produce ambitious collective impacts.

## Conclusion: Approaching health improvement as a governance problem

Our focus on clarity, congruity, and capacity helps streamline the health improvement ‘playbook’ – focused on maximising coherent and effective inter-sectoral action – and relate it to conditions of complexity over which all policy actors have very limited control:

1. *Clarity*. Encourage and exploit a ‘window of opportunity’ to generate high levels of acceptance for health improvement in principle and ‘lock in’ a clear and detailed strategy (backed by regulations, earmarked finance, organisations, and guidance) to secure a lasting mandate.
2. *Congruity*. Align health improvement to existing initiatives to capitalise on synergies and bring service delivery partners ‘on board’.
3. *Capacity*. Nurture critical mass, in terms of the actors working to deliver health improvement objectives, and encourage champions to show leadership and bridge the gaps between levels and sectors.

Each element of this strategy will inevitably face difficulties determined by the political, social, and organisational dynamics of each context. These challenges include: to generate or sustain attention to, and enthusiasm for, health improvement; political or social pressure to prioritise other state activities (including the management of the economy) over health improvement or prevention; and, resistance from key service delivery actors unwilling or unable to change operational procedures or organisational culture. Further, solving all of these issues while avoiding trade-offs – for example between top-down control and bottom-up ownership of, and compliance with, delivery activities – is impossible. Nevertheless, by approaching health improvement as a policymaking problem, it may be possible to identify clearly, and mitigate against, some of the most glaring pitfalls while engaging with inevitable governance dilemmas.

There is no straightforward way to take action on health improvement. In fact, our contemporary context - of unprecedented attention for public health - makes achieving health improvement aspirations more difficult. Attention, resources, and relationships poured into these efforts have been disrupted and displaced by crisis management. In these circumstances, the old HiAP playbook looks unpromising. While a focus on clarity, congruity, and capacity does not solve these problems, it encourages health improvement advocates to maximise their chances by being clear on what they seek to achieve and consistent and realistic in how they seek to achieve it.

## Data availability

No data are associated with this article.

## Ethics and consent

Ethical approval and consent were not required.
