# Supplementary material for: Why is health improvement policy so difficult to secure?
Source: Open Res Eur. 2022 Aug 26;2:76. Originally published 2022 Jun 14. [Version 2] doi: 10.12688/openreseurope.14841.2 (PMC10445925; doi:10.12688/openreseurope.14841.2)
Supplement: Peer review history

# Peer review: Reviewer response for version 2

**DOI:** 10.21956/openreseurope.16281.r29569

**Published:** 2022-09-30

**Authors:** Scott Greer

**Affiliations:** 1 Department of Health Management and Policy, University of Michigan School of Public Health, Ann Arbor, MI, USA

This is a powerful and creative synthesis rooted in policy theory combined with an extensive narrative review of literature on one particular issue, Health in All Policies and its disappointments. The Essay comes in two parts. One is essentially a general theory of public policy which focuses on the interaction between the nature of a policy idea and the political environment in which it exists as a way to explain its adoption and implementation. The theory is the creative, synthetic approach of the three Cs. The case studies are HiAP, as informed by a large narrative review of the literature on the topic complemented by a somewhat more speculative analysis of how to interpret the fate of HiAP in the COVID-19 pandemic. The writing is fluent and there is lots to quote approvingly.

 The weaknesses of the article are drawn from this structure. I think *all *of them could be addressed by light textual refinements and perhaps a clear limitations section that would specify the applicability of the ideas outside the health improvement literature and context in which it receives its empirical content here. The two themes of my comments, which I hope are constructive, is that the discussion downplays the importance of raw politics and shows a strong influence from one particular literature and topic, HiAP, on what seems to be a more general theory. Reading Julia Lynch’s work might be very helpful.

 First, there is the possibility that all of this is cheap talk by governments, knowingly replacing policies that might work with ineffective ones; or knowingly adopting policies that won’t work because no plausible alternative policies exist that might work. HiAP is almost tailor-made for governments to look busy if that is what they need to do, and also to win some support from health advocates who think half a loaf is better than no bread at all. As they note, “Securing high levels of national and international in-principle commitment to a vague idea can represent an alternative to substantive change.” Is lack of clarity, in particular, a structural feature of HiAP or a deliberate feature introduced by policymakers for some reason such as coalition maintenance or reluctance to actually adopt an effective policy?

 Second, the whole of interest group politics and, I think, party politics is to be found in one-half of one C (capacity). The authors are wholly aware of the existence of these powerful forces, but downplay them. This is not a big issue in the HiAP literature they review (since it is mostly about events within government, which means interests are mostly shadowy forces behind what look like noncooperative departments) but it is rather a big issue in the COVID-19 debate, where a variety of interests (e.g. workplaces that did not want to protect customers and workers) and partisan actors (often of the populist radical right) had a big influence on the implementation and adoption of policies. Much prevention policy directly harms somebody’s business and leads to opposition. Is it right to collapse that into one component of capacity? Does the essay need a fourth C of, I don’t know, clout? Otherwise the conclusion that “there only appears to be a high commitment to HiAP when we view that commitment in isolation” doesn’t really have an obvious antecedent.

 Third, HiAP is a very weird policy idea, with a superficial appeal (something for nothing!) and huge problems starting with a willful defiance of everything we know about bureaucracies and a name seemingly chosen to irritate people who work in “all policies.” It disguises all sorts of interests behind what appear to be problems of bureaucratic coordination - a refusal to produce healthy transport systems might have something to do with lobbies for road transport, toll operation, and road building rather than fatheaded bureaucracy in the transport ministry (or, better, that seeming fatheadedness is simply one manifestation of a whole interest constellation). Further, it is primarily evaluated by health promotion and public health analysts with a particular way of thinking and a reluctance to give subtle political explanations for policy outcomes. Insofar as the case is the data validating the text, it needs contextualization of how it demonstrates the main theory; if it’s illustrative, that should be made clear and some discussion of the limits of this example would be helpful. The plain-language summary contains a nice description of the cases that HiAP and COVID-19 responses are; it is not really followed through in the text but could be the basis for a simple categorization of which issues fit well with the general theory and which issues would require using it with different emphases.

 Fourth, the essay underplays the distinction between adoption and implementation. A lot of the HiAP literature is about the relative failure of a plan, reorganization, or budgeting technique. In other words, implementation of something lighter than specific legislation. Does the theory work equally well to explain adoption of anything at all, adoption of the specific instruments (already indicators of government seriousness), and implementation of any specific instrument?

 Finally, the bibliography needs work. I think a software program introduced gremlins; it is particularly hard to identify the authors’ own work correctly.

Is the argument persuasive and supported by appropriate evidence?

Partly

Is the work clearly and cogently presented?

Yes

Is the topic of the essay discussed accurately in the context of the current literature?

Yes

Does the essay contribute to the cultural, historical, social understanding of the field?

Yes

Reviewer Expertise:

Political science, public health, including HiAP at times, in OECD countries.

I confirm that I have read this submission and believe that I have an appropriate level of expertise to confirm that it is of an acceptable scientific standard, however I have significant reservations, as outlined above.

# Peer review: Reviewer response for version 2

**DOI:** 10.21956/openreseurope.16281.r29987

**Published:** 2022-09-05

**Authors:** Tim Tenbensel

**Affiliations:** 1 Associate Professor, School of Population Health, Faculty of Medical and Health Sciences, University of Auckland, Auckland, New Zealand

I'm happy with the changes and clarifications made. Thank you to the authors for such a perceptive and useful synthesis of a large body of work. This will be a great resource.

Is the argument persuasive and supported by appropriate evidence?

Yes

Is the work clearly and cogently presented?

Yes

Is the topic of the essay discussed accurately in the context of the current literature?

Yes

Does the essay contribute to the cultural, historical, social understanding of the field?

Yes

Reviewer Expertise:

NA

I confirm that I have read this submission and believe that I have an appropriate level of expertise to confirm that it is of an acceptable scientific standard.

# Peer review: Reviewer response for version 2

**DOI:** 10.21956/openreseurope.16281.r29986

**Published:** 2022-09-02

**Authors:** Ben Hawkins

**Affiliations:** 1 MRC Epidemiology Unit, University of Cambridge, Cambridge, UK

The authors have engaged with, and responded thoughtfully to, my previous comments. I think the argument is now much clearer and I am happy to recommend indexing it in its current form. My only further (very minor) comments are:

1. There are several one sentence paragraphs in the plain language summary and introduction. Perhaps these could be combined with preceding/subsequent paragraphs?
2. Is the bold text in the final paragraph above the section 2 heading ('The triple threat...') meant to be a heading? This looked a bit incongruous so I suggest maybe removing and integrating the point into the paragraph.

These are just presentational suggestions so feel free to ignore and no need for any further formal revisions or responses.

Is the argument persuasive and supported by appropriate evidence?

Yes

Is the work clearly and cogently presented?

Partly

Is the topic of the essay discussed accurately in the context of the current literature?

Yes

Does the essay contribute to the cultural, historical, social understanding of the field?

Yes

Reviewer Expertise:

Health policy, policy studies

I confirm that I have read this submission and believe that I have an appropriate level of expertise to confirm that it is of an acceptable scientific standard.

# Peer review: Reviewer response for version 1

**DOI:** 10.21956/openreseurope.16031.r29568

**Published:** 2022-07-18

**Authors:** Tim Tenbensel

**Affiliations:** 1 Associate Professor, School of Population Health, Faculty of Medical and Health Sciences, University of Auckland, Auckland, New Zealand

Thank you for the opportunity to review this article. I am very interested in the work of these authors and I have found their contributions on the topic of health improvement and prevention policy to be incredibly useful in both teaching and research. It is great to see this synthesis. I am very sympathetic to the overall argument that there is something about health improvement and prevention policies that is extremely challenging in the context of 21 ^st^ century government, policy and governance. The work of these authors effectively distils a broad body of policy studies literature relevant to the questions they pose.

 Overall, this is already a very strong and well-argued contribution to the literature. The comments and suggestions I have may be useful in sharpening and strengthening it further, hopefully.

1. The article is structured according to three research questions outlined on p3-4. Having looked at the whole article, it wasn’t quite clear to me how Q1 and Q2 relate to each other. There is significant overlap between the content covered in answering these questions such that there seemed to be some repetition in what was covered under the clarity, congruence and capacity headings. Q1 is probably the question that is less clear out of the two. Is this question asking why such policies are not adopted, or is this about implementation? If the latter, how is it different to Q2? If, however, this question is more about non-decision-making, perhaps that could be made clearer.
2. What is the relationship between the key terms ‘prevention’, ‘health improvement’ and ‘health-in-all policies’? Are they all synonymous with each other? Or is one of them an overarching term of which the others are subsets? Or is the problem that all these terms are used, but each have somewhat different ambits? I ask this because some of the points made in the article may apply more directly to some but not all of these terms. Health improvement, for example, generally covers what happens in health services as well as broader social determinants and inter-sectoral aspects of health.
3. The discussion about clarity on p5 is very insightful. I couldn’t help wondering whether the issue about clarity is about the definition of problems or of solutions or both. Much of the discussion in these paragraphs seems to be about the lack of clarity around problem definition. However, it could be argued, a la Lindblom that this doesn’t really matter as long as there is some broad agreement about particular solutions. So if we take Sure Start as an example, many government agencies and interest groups may support Sure Start for many different reasons – many of which have little to do with health improvement. As long as the ‘programme’ is reasonably clear, does fuzziness about the problem(s) meant to be solved really matter? Regarding obestity, we have a rather different dynamic in which there is agreement that ‘something needs to be done’, but little agreement about what. So where precisely is the challenge around clarity – in problem definition or solution specification?
4. I think the article works best when discussing the challenges of inter-sectoral and health-in-all approaches to prevention and health improvement (of which Sure Start seems to be the most tangible example). For this type of initiative, the arguments about clarity, congruity and capacity work very well. However, the authors seem to be referring to more ‘traditional’ public health policy concerns in the section on p6 under ‘capacity’. Here the power of industry is discussed, and this seems most relevant to debates about accessibility, advertising and pricing of alcohol, junk-food, tobacco and gambling (what some refer to as the commercial determinants of health). In these types of issues, is it really capacity that is lacking, or is it power, plain and simple? States do have plenty of regulation capacity, but they are often not willing to use it in the face of real and anticipated industry opposition. At the very least, these are very different types of capacity constraints to those faced by inter-sectoral policy initiatives such as Sure Start.
5. For Question 3, what exactly is the irony referred to? Some may argue that a focus on protection is inevitably in conflict with a focus on prevention as public health policy resources and capacities are inherently limited. A broader question about COVID-19 and the relationship between protection and prevention is whether the things that the authors are referring to are generalizable or specific to country contexts. In my own country, both agendas are playing out simultaneously.
6. Another point made under the answer to Q3 is the tension between top-down, hierarchical stipulation and allowing room for bottom-up collaborative experimentation. This seems to be a much broader point about a fundamental tension of governance in any policy area. Perhaps it is not surprising that we would find advocates within public health for both approaches. Are they the same advocates? Is this substantively different or unique to what is happening in other policy areas? Or is there something particularly peculiar about the way this governance tension plays out in health prevention and improvement?

Is the argument persuasive and supported by appropriate evidence?

Yes

Is the work clearly and cogently presented?

Yes

Is the topic of the essay discussed accurately in the context of the current literature?

Yes

Does the essay contribute to the cultural, historical, social understanding of the field?

Yes

Reviewer Expertise:

Health policy, public policy, policy implementation

I confirm that I have read this submission and believe that I have an appropriate level of expertise to confirm that it is of an acceptable scientific standard.

# Response

**Published:** 2022-08-22

**Authors:** Paul Cairney

**Affiliations:** University of Stirling, UK

Thank you for your thoughtful and constructive review. In line with your comments, we have made the following changes, largely for the sake of clarity:

 1. Clarify the three main research questions, and 2. Clarify the meaning of key terms - ‘prevention’, ‘health improvement’ and ‘health-in-all policies’ – and how they relate to each other. *We reframed these questions (in the introduction) to provide a clearer way to distinguish between key terms*.

 3. Explain that ambiguity relates to unclear problem definition, which resurfaces as a problem when policy actors seek specific solutions. *We added a paragraph to this section to describe ambiguity in relation to problem definition and solution proposing*.

 4. Clarify that our explanations may relate differently to different examples: some are primarily about joining-up policymaking, while others are about power. *We made this point much more clearly in the capacity section, separating the Sure Start and industry examples*.

 5. Consider if the COVID-19 experience is really ironic (isn’t a conflict between protection and improvement inevitable rather than unexpected?) and if our points are generalizable. *We remove reference to irony*. *We use the WHO 2020 report to highlight a general shift from improvement to protection (while qualifying the language to describe ‘many countries’)*.

 6. Consider if the tension in governance, that we describe, is a general feature of policymaking or if it plays out in distinctive ways in public health. *We clarify (albeit briefly) that we are describing a general effect that is discussed frequently in HiAP reports.*

# Peer review: Reviewer response for version 1

**DOI:** 10.21956/openreseurope.16031.r29570

**Published:** 2022-07-07

**Authors:** Ben Hawkins

**Affiliations:** 1 MRC Epidemiology Unit, University of Cambridge, Cambridge, UK

The article seeks to explain the enduring gap between rhetorical commitments to health prevention and its successful translation (and implementation) into policy outputs using relevant concepts from political science and policy studies (i.e. bounded rationality, policy complexity). As such, it address an important real world problem and one which has presented both a conceptual and empirical challenge to health policy actors and researchers. The attempt to deepen understanding through the application of cross-disciplinary theoretical insights is to be welcomed. The authors do so by identifying 3 key factors – clarity, congruity and capacity – as explaining the prevention ‘gap’ in health policies, which are applied to analyse health in all policies and covid 19 responses as policy case studies.

 While the first and the third of these are intuitively plausible factors, congruity, and the mechanisms through which they impact on policy are set out in relation to previous studies, the concept of congruity, and its explanatory power in this framework, are less clearly explained. It could be argued that a lack of congruity between rhetorical commitments to prevention and the capacity of the policy-making architecture to deliver this restates the problem that the article seeks to explain rather than identifying characteristics of the policy process that account for this. I think this section (and the relevant passages of the introductory and background sections introducing the concept), therefore, need to be revised to clarify precisely what is meant by congruity and what the specific insights to be gained from this concept are. What it seems to be about is the inability of sedimented policy systems to facilitate ‘radical’ policy change? But this seems to be less of an issue of congruity with health prevention than a more general issue of path dependence and institutional memory and the default to the status quo? Another issue is how this interacts with issues of political will to force change despite this inertia. Can the issue of committed policy entrepreneurs be completely set aside as the introduction seems to suggest?

 Related to this, I think that the concept of radical policy change needs to be more clearly introduced and explained. Does this simply mean doing prevention effectively or is it a specific model of prevention (for example, on page 5, column 2, 1st paragraph, it is unclear if ‘radical advocates’ are the same as ‘proponents in public health’ or they hold two different positions)?

 The article could also benefit from some restructuring and material being moved elsewhere. The first paragraph of section 1 reads more like introductory material to me. This would be useful as the introduction at present doesn’t set out clearly the main focus and argument of the article. I suggest revising so that it frames the article more clearly for the reader.

 Related to this, the methods section seems to contain material that belongs more logically elsewhere while not really explaining completely clearly what was done. I suggest you consider moving the material in paragraph 1 to the introduction and adding an additional paragraph on how the article was conceived and executed. Paragraph 2 reads like a summary of the argument or introduction to the main argument and could go in the intro or as an opening to the main sections (currently labelled 1, 2, 3).

 On page 5 the point about temporary agreement: is it temporary agreement or people just talking about 2 different things using the same label? I’m not sure that it is primarily about temporality so I suggest rephrasing. This paragraph also seems to contain important issues – contests over meaning and to define policy issues – which could be further unpacked and explored as reasons for lack of clarity and the prevention gap. Engagement with the literature on policy framing could assist. Are there citations or evidence for the final sentence?

 The case studies are well presented and explained and the inference drawn about the specific findings on these and about health policy are generally are well made.

**Minor points:**

 Abstract:

- Line 3, change to ‘…policy statements on the one hand and practices and outcomes on the other.’?
- Health prevention and improvement are both used. Are these the same in your view or do they differ? Perhaps use just one here and/or define the terminology in the main article.
- Line 12, health protection ‘seeks to’ inoculate.
- Are they just (as opposed to primarily) about NCDs or as covid showed is it also about susceptibility to other morbidities including infectious diseases?
- Line 18, delete colon.
- Not sure what ‘tethered to political reality’ means. Rephrase/clarify.
- Following line change ‘always’ to 'in general’ or something slightly less absolute/categorical.
- 2 lines later ‘a tendency’ amongst whom?
- Next line: systemic policymaking dynamics is a bit unclear. Could this be reworded? Maybe ‘characteristics of the policy-making system’?

Plain language summary:

- This leads with covid but the article is not principally about this. The meaning of the first sentence is also unclear.
- Is ‘social determinants of health’ understood without explanation to non-specialists? Likewise the idea of these being ‘distributed evenly’ and the protection prevention distinction?
- Second paragraph: The opposite of what?
- Third paragraph, sentence 2: the logic is unclear. Next sentence: If these gaps are inevitable why bother trying to reduce/end them? Rephrase.
- Final paragraph is unclear to me.

Main text:

- I found the numbering of the main sections of the article confusing and suggest deleting or editing to reflect their position in the text.
- P4 column 1 last paragraph before ‘clarity’ heading. Has a long complex sentence that was unclear. Rephrase?
- Heading ‘interpreting covid in that old context’ doesn’t sound quite right. Rephrase?
- Next heading: should it be ‘the’ (not ‘a’) covid 19 era?

Is the argument persuasive and supported by appropriate evidence?

Yes

Is the work clearly and cogently presented?

Partly

Is the topic of the essay discussed accurately in the context of the current literature?

Yes

Does the essay contribute to the cultural, historical, social understanding of the field?

Yes

Reviewer Expertise:

Health policy, policy studies

I confirm that I have read this submission and believe that I have an appropriate level of expertise to confirm that it is of an acceptable scientific standard, however I have significant reservations, as outlined above.

# Response

**Published:** 2022-08-22

**Authors:** Paul Cairney

**Affiliations:** University of Stirling, UK

Thank you for your thoughtful and constructive review. We have summarised your comments and made the following changes:

 1. Clarify the meaning of ‘congruity’ throughout. *We added a discussion of the multiple ways to describe the forces of inertia or continuity that come under our general banner of congruity*.

 2. Clarify the concept of radical policy change in relation to preventive public health and advocates for radical policy change. *We clarified this term when it first arises (in ‘The general problem with prevention policies’), and in relation to the specific example of obesity*.

 3. Consider some restructuring of the essay. For example, consider moving the 1 ^st^ paragraph of section 1 to the introduction to improve the latter. *We kept this section in the introduction, but used it as a source to help improve those earlier sections*.

 4. Consider moving some of the methods section (paragraph 1) and replacing it with a more straightforward discussion of the article’s conception and execution. *We moved most of the text to the Introduction, and tightened up the discussion of our approach*.

 5. Clarify the paragraph on temporary agreement (and provide a citation for the final sentence). *We largely keep this paragraph as is. We describe the temporary appearance or feeling of agreement that relates to ambiguity (in other words, we describe people using the same language to mean different things). Temporality is key, since we describe an initial feeling of agreement in broad principle replaced by disagreement in practice*. *We clarify the referencing at the end of the paragraph (and add more reference to framing earlier on).*

 6. Address a series of minor points, including to clarify the differences between general preventive policies and specific strategies (Abstract and main text), rephrase the Plain language summary, and attend to some confusion in the Main text. *We made most of these suggested changes, and performed an additional check for clarity throughout the essay*.
